# Melting of AuPd Nanoparticles Revisited: Geometry and Size Effects

**DOI:** 10.3390/ma18051054

**Published:** 2025-02-27

**Authors:** Andrés Soria-Sánchez, Miguel Angel Rayas, Antonio Ruiz-Aldana, Juan Andrés de la Rosa-Abad, Sergio Mejía-Rosales

**Affiliations:** 1 Dimex Capital, Monterrey 64650, Mexico; 2 Facultad de Ciencias Físico-Matemáticas, Universidad Autónoma de Nuevo León, San Nicolás de los Garza 66455, Mexico; mrayasx@uanl.edu.mx (M.A.R.); antonio.ruizald@uanl.edu.mx (A.R.-A.); juanderoab@unc.edu.ar (J.A.d.l.R.-A.); 3 Centro de Investigación en Ciencias Físico-Matemáticas (CICFIM), Facultad de Ciencias Físico-Matemáticas, Universidad Autónoma de Nuevo León, San Nicolás de los Garza 66455, Mexico

**Keywords:** AuPd nanoalloys, twin boundary effects in nanoparticles, order parameters in nanoparticle melting, HAADF-STEM simulations of nanoparticles

## Abstract

The thermal stability of bimetallic nanoparticles plays a crucial role in their performance in applications in catalysis, biotechnology, and materials science. In this study, we employ molecular dynamics simulations to investigate the melting behavior of Au-Pd nanoparticles with cuboctahedral, icosahedral, and decahedral geometries. Using a tight-binding potential, we systematically explore the effects of particle size and composition on the melting transition. Our analysis, based on caloric curves, Lindemann coefficients, and orientational order parameters, reveals distinct premelting behaviors influenced by geometry. Larger particles exhibit a coexistence of a pseudo-crystalline core and a partially melted shell, but, in decahedra and icosahedra, melting of the core occurs unevenly, with twin boundaries promoting the melting of one or two of the tetrahedral subunits before the rest of the particle. Notably, icosahedral nanoparticles display higher thermal stability, while both icosahedral and decahedral structures exhibit localized melting within twin boundaries. Additionally, we generate HAADF-STEM simulations to aid the interpretation of in situ electron microscopy experiments.

## 1. Introduction

Metal nanoparticles are one of the most studied types of nanostructures, mainly because of their applications in fields such as catalysis, biotechnology, and electronics [1]. In the biomedical sector, metal nanoparticles are used for drug delivery, imaging, and as therapy agents [2,3,4]. They are also employed in catalytic reduction reactions, oxidation-reduction processes, and reforming reactions [5], having a considerable impact in activities as important as pollution control, and selective hydrogenation reactions in food and oil industries [6]. It is understandable then that different approaches are used to improve our understanding of the thermodynamic properties of these nanostructures, since this is relevant for fine-tuning their potential applications, particularly in high-temperature environments.

The use of bimetallic nanoparticles opens an even wider window of opportunity, since the appropriate selection of the two chemical elements may produce synergistic effects. It has been proposed that in bimetallic nanoparticles the electron transfer between the two components significantly contribute to their improved performance as catalysts Zhang et al. [7]. In particular, Au-Pd metallic nanoalloys are highly efficient catalysts for trichloroethene (TCE) degradation [8], and have been employed as catalysts for the hydrogenation of naphthalene and toluene, which are important reactions in the enhancement of diesel fuels.

In all of these applications, having the ability to predict –or even control– the melting temperature of the nanoparticles becomes a critical factor, for several reasons. First, keeping the system below the melting point reduces agglomeration, and ensuring that the nanoparticles remain below their melting temperature preserves their original geometries; second, controlling the melting temperature ensures the nanoparticles remain active and stable as catalysts; third, in bimetallic nanoparticles, controlling the melting temperature is essential to achieve the desired alloy composition and phase structure. It is worth noting that most synthesis methods for Au-Pd nanoparticles (alcohol reduction, the sonochemical method, citrate reduction, vacuum vapor deposition technique, and reverse micelles) involve heating at some stage [1].

The use of molecular dynamics (MD) simulations is a highly effective method for investigating the structural properties and thermodynamic evolution of metal nanoparticles. For instance, the hysteresis behavior in caloric curves of Ag nanoparticles has been examined using this approach [9], and it has been well-established that metal nanostructures exhibit lower melting points than the same material at bulk [10], as predicted by numerous MD simulations [11,12,13], corroborated by experimental studies [14], and explained in terms of thermal phonon contribution [15] and nanothermodynamics [16]. Geometry and size are also critical factors in the appearance of the melting transition, as it has been explained by Barnard et al. [17]; here, they use relativistic ab initio thermodynamics to show that changes in Gibbs free energy of formation favors different motifs in Au nanoparticles depending on size: from icosahedral to decahedral, and then to truncated octahedral as size increases. Similarly, Myshlyavtsev and Stishenko find that icosahedral shapes are more stable at smaller sizes, while cuboctahedral shapes become more stable as size increases [18]. Mejía-Rosales and Fernández-Navarro [19] analyzed the cooling process of Au-Pd nanoparticles, finding that the transition led to the formation of well-defined low-energy icosahedral structures and other geometries with an FCC environment interspersed with bands of HCP atoms. Taking all of this into consideration, one of us worked on investigating the melting transition of AuCu icosahedral, decahedral, and cuboctahedral nanoparticles using MD, finding surface premelting and partial melting phenomena influenced by the specific geometry and composition, with decahedral nanoparticles showing significant surface rearrangements close to the melting transition [12].

In a previous article, this group worked on the investigation of the melting transition of bimetallic Au-Pd nanoparticles using MD simulations; the study focused on the structural properties of cuboctahedral nanoparticles at several relative compositions during a simulated heating process, and the two-stage melting dynamics of the Au-Pd nanoparticles was described. Due to theoretical and infrastructure limitations, this work was somewhat narrow both in scope and methodology: it dealt with a single geometrical motif, small particle sizes, and a limited set of compositions. Interactions were modeled using the Rafii-Tabar and Sutton version of the Sutton and Chen interatomic potential, that was the better choice to model fcc alloys at the time; while this potential is still used due to its simplicity and computational efficiency [12,20,21], newer potentials, such as those derived from Density Functional Theory and/or Machine Learning, offer significantly improved accuracy. In this paper, we revisit the melting transition in Au-Pd nanoalloys, focusing in nanoparticles with three particular geometries that frequently appear in this kind of metal nanoparticles at the studied scales: decahedral, icosahedral, and cuboctahedral. This study is somewhat informed by the paper due to Martínez-Muñoz et al. [12], that deals with the melting transition in Au-Cu nanoparticles with these geometries. In the case of Au-Pd nanoparticles, it has been shown both experimentally and theoretically that spontaneous appearance of defects in the atomistic arrangements may help to stabilize the particles to take icosahedral, decahedral, and cuboctahedral shapes [22], making these geometries particularly interesting for our investigation.

While direct experimental evidence is scarce of the depletion of melting in metal nanoparticles, there have been some efforts that are worth to mention. Heating and melting dynamics of gold nanoparticles in water has been investigated using time-resolved X-ray scattering, finding that the melting point of nanoparticles is significantly lower than bulk gold, and pre-melting effects are observed before reaching the melting temperature [23]. Gafner et al. studied the heat capacity and melting behavior of fcc metals, including palladium, and observed deviations in the melting points for nanoparticles compared to bulk materials [24]. These results are consistent with theoretical approaches [17].

The main objective of this study is to analyze the dependence of the melting transition on the structure and composition of bimetallic Au-Pd nanoparticles. We implement MD simulations of the heating process, and use specific order parameters to characterize the solid-liquid transition. The paper is organized as follows: The second section describes the details of the simulation and the analysis methods, including the interatomic potential used for the simulations and the order parameters calculated to determine the melting line for each structure. The third section presents the main results obtained by the simulations, while in the final section we discuss the results under a perspective that might be of interest from an experimental design standpoint.

## 2. Methods

We employed molecular dynamics (MD) simulations to study and characterize nanoparticles with specific geometrical features. Specifically, we focused on decahedral, icosahedral, and cuboctahedral Au-Pd nanoparticles using a tight-binding potential [25] to describe the interatomic interactions. The specific set of parameters for the potential, due to Pittaway et al. [26], was chosen to enable comparison with other studies and to evaluate its performance and reliability. To characterize our results, we utilized a series of well-established order parameters, such as the Lindemann coefficient [27], and $Q_{6}$ order parameter.

A many-body potential such as the Pittaway et al. version of the Gupta potential used in this study accounts for both pairwise interactions and many-body effects, which are crucial in modeling the cohesive energy and structural properties of metal alloys at the nanoscale. The parametrization due to Pittaway reproduces experimental data, such as lattice constants and cohesive energies [28], making it well-suited for accurately simulating the structural and dynamic behavior of nanoalloys, where surface and size effects play a significant role. This potential is able to consider the relative stability of icosahedral, decahedral, and fcc-like structures in Au-Pd clusters, unlike some alternative many-body potentials that may underemphasize these structural motifs [29]. It also produces a more reliable prediction of Au-Pd mixing behaviors at various compositions and temperatures [26].

**Figure 1:**
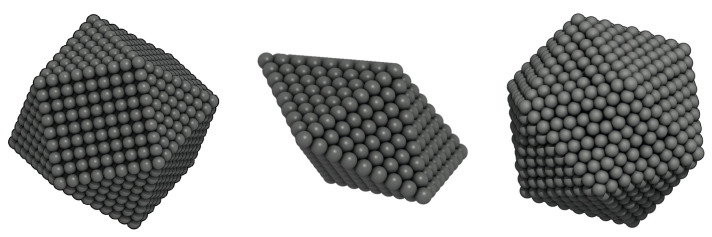
Representations of the three geometries of the nanoparticles considered in this study. (**left**) Cuboctahedron; (**center**): Decahedron; (**right**) Icosahedron.

The MD simulations were performed using the LAMMPS code, release of 2 August 2023 [30]. Ten initial structures were prepared, three with cuboctahedral geometry (2057, 6525, and 12,431 atoms), three with icosahedral geometry (2057, 6525, and 12,431 atoms), and four with decahedral geometry (609, 1111, 1833, and 4097 atoms). While the decahedra and cuboctahedra were easily obtained directly from an fcc arrangement, the case of icosahedra requires a modification of the fcc lattice: in the tetrahedra that form each icosahedron, the fcc structure must be distorted in such a way that the distance between first neighbors within the same layer–icosahedra have an onion-like structure—is 1.05 times the distance between first neighbors in the same layer, ensuring the tetrahedra form a closed icosahedron [31]; we took this into consideration for the creation of the initial icosahedral configurations. A representation of the three geometries is shown in Figure 1.

The simulations were conducted within the canonical ensemble using a Nosé-Hoover thermostat to control temperature. Periodic boundary conditions were applied in all three dimensions, but bulk-like behavior was avoided by centering the nanoparticles inside a simulation box around three times their diameter. Following the approach of Bertoldi et al. [32,33], initial conditions were prepared by performing an energy minimization to ensure the molecular dynamics (MD) simulations started from stable configurations, and subsequently a temperature ramp was applied to the system, where the temperature was increased incrementally by 25 K every 200 ps, using a time step of 0.001 ps. At each temperature increment, an additional 200 ps run was performed: the first 100 ps were allocated for system equilibration, and the subsequent 100 ps were used for data collection. The equations of motion were integrated using the Velocity-Verlet algorithm. The simulation sequence was terminated once the system temperature reached 1200 K. The File S1 of the Supplementary Materials contains the LAMMPS script for the whole simulation process. The data collected during the heating process was subsequently analyzed to calculate order parameters and to study the structural and dynamic properties of the system.

The melting process of the nanoparticles was analyzed by examining the MD-derived caloric curves, which plot the potential energy per atom as a function of temperature. These curves allow us to track the abrupt energy changes associated with the enthalpy of fusion, such that a significant change in the slope of the caloric curve is indicative of the melting transition [34]. This approach enables an identification of a narrow range of temperatures at which melting takes place but, since melting may not occur in the particle as a whole at an specific temperature, we also used the Lindemann parameter, related to the root-mean-square bond fluctuation [35]. The Lindemann parameter for each atom was calculated as [27]

(1) $$\delta_{i}=\frac{1}{N-1}\sum_{j(\neq i)}\frac{\sqrt{{\langle r_{\mathrm{ij}}^{2}\rangle }_{T}-{\langle r_{\mathrm{ij}}\rangle }_{T}^{2}}}{{\langle r_{\mathrm{ij}}\rangle }_{T}}\;.$$

In Equation (1), *N* is the number of atoms in the cluster, ${\langle \ldots \rangle }_{T}$ represents the ensemble average at the temperature of the sample, and $r_{\mathrm{ij}}$ is the separation distance between atoms *i* and *j*. The overall Lindemann index was calculated by taking the average to the Lindemann parameter for each atom [32]. The Lindemann parameter has a larger value in liquid state than in solid state [36], which helps us identify the melting of the nanoparticles in a complementary way to the use of caloric curves.

Local ordering was studied considering the bonds between neighboring atoms separated a distance smaller than a radius $r_{\mathrm{cut}}$. The orientation of the bonds $r_{\mathrm{ij}}$ was used to calculate the individual spherical harmonics $Y_{\mathrm{lm}}\left(r_{\mathrm{ij}}\right)=Y_{\mathrm{lm}}\left(\theta_{\mathrm{ij}},\phi_{\mathrm{ij}}\right)$ [34]. In particular, we set l=6, and for each atom the spherical harmonics were averaged over all its bonds:

(2) $$q_{6m}\left(i\right)=\frac{1}{N_{n}\left(i\right)}\sum_{j=1}^{N_{n}\left(i\right)}Y_{6m}\left(r_{\mathrm{ij}}\right)\;,$$

where $N_{n}\left(i\right)$ is the number of neighbors of the *i*th atom. The average $q_{6}$, defined as

(3) $$q_{6}\left(i\right)={\left[\frac{4\pi }{2l+1}\sum_{m=-6}^{6}{\left|q_{6m}\left(i\right)\right|}^{2}\right]}^{1/2}$$

can be used as a quantitative measure of the local sixfold order around an atom: values close to 0.575 are evidence of FCC structure, 0.485 for HCP structure, and 0.663 for icosahedral-like structure [37]. The global order parameter $Q_{6}$, as presented in Equation (4), takes different values depending on the kind of crystal structure in a solid, and for melted states its value drops down close to zero [12,38]. Here, $Q_{6m}$ is the average of $q_{6m}$ along all of the bonds in the nanostructure:

(4) $$Q_{6}={\left(\frac{4\pi }{13}\sum_{m=-6}^{6}{\left|Q_{6m}\right|}^{2}\right)}^{1/2}\;.$$

The definition of a local order parameter enabled us to map the melting process within the volume of the nanoparticle. In order to verify that the behavior of the order parameters is not strongly dependent on the specific initial conditions of the structures, for each nanoparticle the simulated heating process was repeated using at least three different initial velocity distributions. Since no relevant differences were found, we can trust that the curves that we are reporting here are thermodynamically stable.

In order to offer a fair comparison against experimental observations, for a selection of configurations we obtained High-Angular Annular Dark Field Scanning Transmission Electron Microscopy (HAADF-STEM) micrographs. Knowing from previous experience that historically the real simulation of atomistic STEM (this is, the simulation of the interaction of the electron beam with the atoms of the sample) had been so computationally expensive that it had been limited to cases of periodic systems or clusters made by a small number of atoms, our first approach was to use an approximation technique that considers the effect of a particular atom in the STEM image through a Gaussian spot, approximation that has been used in the past as an affordable way to obtain STEM simulated images qualitatively useful [39,40]. In this study we took advantage of the availability of Prismatic v2.0, a greatly efficient code for the generation of quantitatively correct simulated HAADF-STEM images [41] Using as input the atomistic coordinates of a particular configuration, and assigning an overall Debye-Waller factor of 0.13 Å, the images were obtained using a beam of 200 kV, third-order aberration $C_{s}=0$, fift order aberration $C_{5}=-0.005$ cm, and a beam convergence 0.01 rad. With these settings, sub-Angstrom resolution was achieved.

## 3. Results

We monitored the average configurational energy of the particles at each temperature considered in the study; we used the results to built a set of caloric curves. We also calculated the average Lindemann coefficient per particle, and the $Q_{6}$ global parameter, for each of the different structures, compositions, and sizes. Since the simulation results for the three geometries considered in this study are independent from each other, we will present them separately in the next subsections.

### 3.1. Cuboctahedra

**Figure 2:**
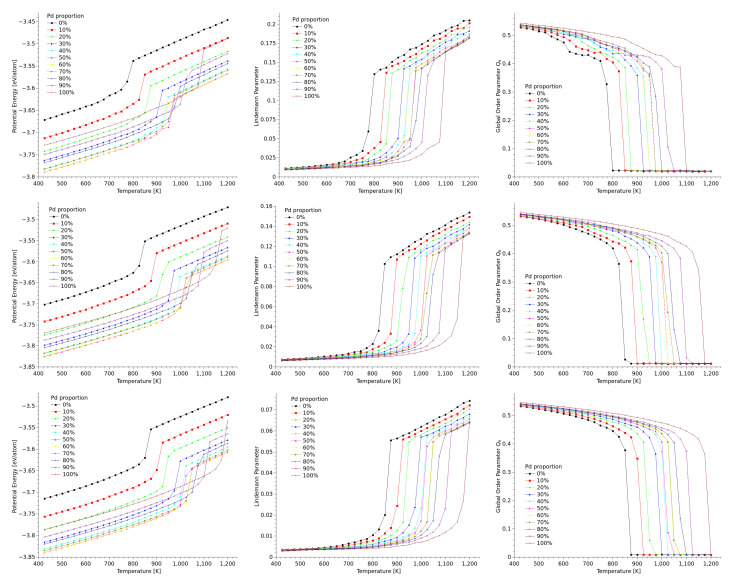
Caloric curves (**left**), Lindemann parameter (**center**), and $Q_{6}$ global order parameter (**right**) as function of temperature for cuboctahedral nanoparticles of 2057 atoms (**first row**), 6525 atoms (**second row**), and 12,431 atoms (**third row**).

The graphs for configurational energy, $Q_{6}$, and Lindemann parameter as function of temperature for cuboctahedral nanostructures of 2057 atoms are presented in Figure 2. The different set of data are associated to different relative compositions of palladium in the structure: for example, the curve labeled as 0% corresponds to an ${\mathrm{Au}}_{100}-{\mathrm{Pd}}_{0}$ structure, and the 30% curve stands for the ${\mathrm{Au}}_{70}-{\mathrm{Pd}}_{30}$ nanostructure. In all of the graphs, the sharp change in the behavior of the curves evidently denote the melting transition, but, more importantly, in all of the graphs it can be noted that the behavior of the value of the measured quantity shifts away from linearity even before reaching the melting transition. This trend is not as evident in the caloric curves as in the Lindemann parameter plots, which denotes that at these temperatures there is only a slight energy investment in the breaking of bonds. Comparing the graphs of $\delta_{i}$ for the three different sizes, the effect of the size of the nanoparticles can be noted straightforward: In the nanoparticles, a significant fraction of the atoms is located at the surfaces, where the coordination number is low and thus the bonding is weaker, which increase the atomic vibrations; this has the effect of increasing the value of the Lindemann parameter as the particle size decreases.

**Figure 3:**
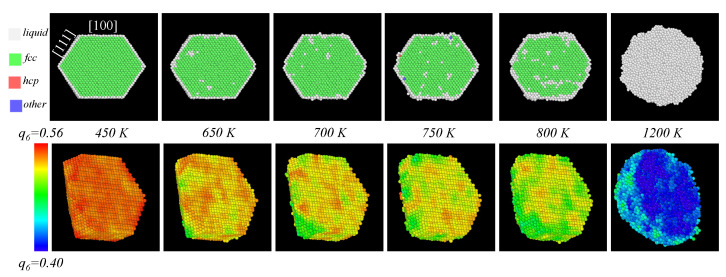
(**upper row**) Sequence of configurations for a sliced 12,431-atom cuboctahedron, where the atoms are colored according to their coordination; note that when the temperature has reached 800 K, the thickness of the premelted surface is larger in [100] faces than in [111] faces. (**lower row**) Sequence of configurations for a sliced 12,431-atom cuboctahedron, showing the evolution of the local orientational order parameter $q_{6}$ as the nanoparticle is heated.

An issue that makes particularly interesting the case cuboctahedral particles is that cuboctahedra have two kinds of atomic planes on their faces, namely [100] and [111]. Melting in metal nanoparticles is commonly driven by surface premelting [42,43], and, as it was well stablished by Dai et al. [44], surface energy anisotropy produces that [100] and [111] surfaces to melt differently: in general, the solid-vapor surface energy $\gamma_{\mathrm{SV}}$ is higher than solid-liquid and liquid-vapor energies, which makes surface premelting thermodynamically favourable but, for [111] surfaces, $\gamma_{\mathrm{SV}}$ is lower than in [100] surfaces, and thus a thinner premelted surface is expected in the former. In our simulations of cuboctahedra this is indeed the case, but measuring this effect in small nanoparticles is quite difficult. In the larger cuboctahedra considered in this study it was possible to differentiate the melted surface from the inner ordered structure of the nanoparticles using coordination analysis and, as an example, we show in the upper row of Figure 3 several snapshots of a 12,431-atom ${\mathrm{Au}}_{80}-{\mathrm{Pd}}_{20}$ cuboctahedron close to the melting transition, where the differences in width of the premelted surface in [100] and [111] surfaces (at 800 K) is evident.

Neither the caloric curves nor the global order parameters by themselves are enough to make a more detailed description of how the structure of the nanoparticles evolves before melting; to correlate this behavior against structural changes we used the local order parameter $q_{6}$. The lower row of Figure 3 shows a sequence of images of the largest cuboctahedron considered in this work, where the color of the atoms were assigned on function of the local average value of $q_{6}$; we choose the composition ${\mathrm{Au}}_{80}-{\mathrm{Pd}}_{20}$ as an example, since the features of the $q_{6}$ appear not to be dependent on the composition of the particle. The sequence images correspond to several configurations as the particle gets heated. For the $Q_{6}$ global parameter, the tendency to reach a zero value after the phase transition is consistent with its definition [34].

### 3.2. Decahedra

**Figure 4:**
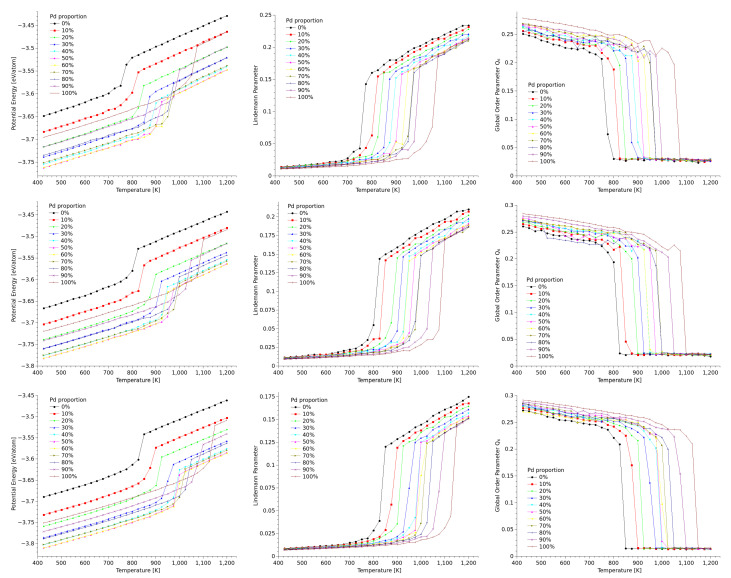
Caloric curves (**left**), Lindemann parameter (**center**), and $Q_{6}$ global order parameter (**right**) as function of temperature for decahedral nanoparticles of 1111 atoms (**first row**), 1833 atoms (**second row**), and 4097 atoms (**third row**).

The synthesis of decahedral Au and/or Pd nanoparticles has been widely reported [45], and it is understood that these and other multiply twinned particles appear as a result of an interplay between surface energy minimization and distribution of internal strain [46,47]. Synthesis of multiply twinned particles is relatively straightforward, either by the heating of aqueous PVP/${\mathrm{HAuCl}}_{4}$ solution, with poly(vinyl pyrrolidone) (PVP) used as a reduction agent [47], by the usual Polyol method [46], or by physical methods, such as inert gas condensation [48], a technique that has produced AuPd nanoparticles with no core-shell distribution. Similarly to the approach used with the simulation of cuboctahedral nanoparticles, in our simulations of decahedra the potential energy was monitored along the heating process, and both the Lindemann parameter and the $Q_{6}$ parameter were calculated. Figure 4 presents the graphs that compare the behavior of potential energy, $\delta_{L}$, and $Q_{6}$ as temperature increased for the different sizes and concentrations considered in the study. Regardless of the geometry, the melting transition can be easily identified using the caloric curves, as shown in Figure 4. However, for further confirmation, the Lindemann parameter provides a clearer indication, as seen in Figure 4, where $\delta_{L}$ exhibits a more abrupt change in slope whenever the melting transition occurs.

**Figure 5:**
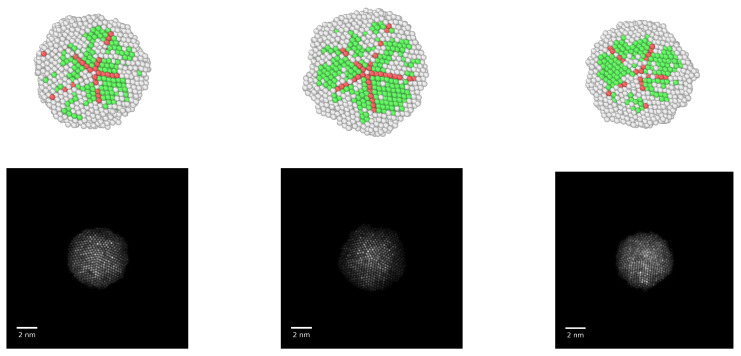
(**first row**) Atomistic representation of three nanoparticles of 4987 atoms, with compositions (**left**–**right**) ${\mathrm{Au}}_{100}-{\mathrm{Pd}}_{0}$, ${\mathrm{Au}}_{50}-{\mathrm{Pd}}_{50}$, and ${\mathrm{Au}}_{0}-{\mathrm{Pd}}_{100}$. The configurations correspond to the temperatures immediately below the melting transition. Colors are associated with the kind of local ordering: fcc (green), hcp (red), and liquid-like (white). (**second row**) Simulated HAADF-STEM micrographs for the same configurations.

Unlike the case of cuboctahedral nanoparticles, where the whole of the volume of the particle—at least in our models—consists in a unique fcc arrangement, in decahedra the twins that delimitate the five tetrahedra play a determining role as the particle melts. Structurally, the twins are grain boundary junction (GBJ) disclinations, and the strain energy associated with these defects make the decahedron a stable structure just at small particle sizes [39]. The effect of the twins on the way melting occurs can be directly observed in the MD dynamical trajectories as temperature increases until almost reaching the melting transition: atomic diffusion starts first in one or several of the tetrahedra, making the melting of the inner volume of the nanoparticle uneven. While surface premelting is still noticeable in decahedral nanoparticles, the twins that act as boundaries in the inner volume of the particle become regions of high atomic instability as temperature is risen, which energetically competes with the relatively stable {111} facets at the surface to become the starting regions for melting. While this would be more easily noted in larger particles than those modeled here, it can still be found in the largest decahedral nanoparticles considered in this study. Figure 5 shows this phenomenon for three nanoparticles of 4987 atoms, with compositions ${\mathrm{Au}}_{100}-{\mathrm{Pd}}_{0}$, ${\mathrm{Au}}_{50}-{\mathrm{Pd}}_{50}$, and ${\mathrm{Au}}_{0}-{\mathrm{Pd}}_{100}$. The individual atoms are visualized in the first row of the figure as colored spheres, where each color corresponds to a particular atomistic arrangement: green for fcc, red for hcp (typically in the grain boundary junctions), and white for amorphous, liquid-like structure. The configurations were chosen close to the melting transition and, as it can be noted, the presence of the liquid phase is distributed either at the surface of the nanoparticle, or concentrated in one or two of the original tetrahedra forming the nanoparticle. In this representation, only one half of the nanoparticle is shown in order to visualize the inner structure of the particles; in the lower row of the figure, we included HAADF-STEM simulated micrographs of the nanoparticles in the configurations shown in the upper row.

A comparison between the ball-and-stick representations and the simulated micrographs show that, even that a considerable volume of the particle has melted, both at the surface and mainly at some of the tetrahedra, the remaining fcc structure still generates bright spots in the micrographs that corresponds with the positions of atomic columns that still remain even in regions where some of the volume is not structured as fcc. Nevertheless, these bright spots are not as intense as those where the volume of the tetrahedra keeps their original crystal structure, even that in these regions the electron beam is interacting with a large amount of material, unlike the regions close to the borders of the particle where the STEM signal is weaker both because of surface premelting, and a relatively small volume interacting with the beam [49]. This interplay between thickness, atomistic ordering, and composition, makes the interpretation of real HAADF-STEM micrographs difficult, which underscores the convenience of using simulated micrographs performed on models where these three features are completely characterized.

### 3.3. Icosahedra

**Figure 6:**
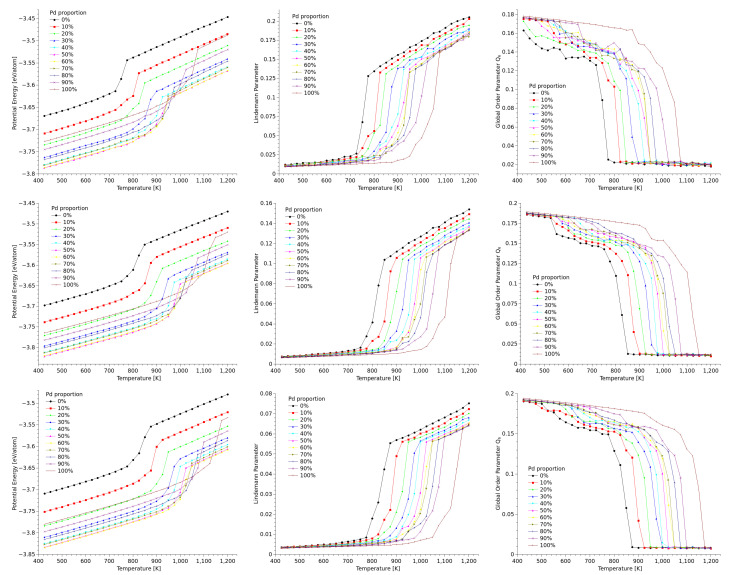
Caloric curves (**left**), Lindemann parameter (**center**), and $Q_{6}$ global order parameter (**right**) as function of temperature for icosahedral nanoparticles of 2057 atoms (**first row**), 6525 atoms (**second row**), and 12,431 atoms (**third row**).

As in the previous sections, the caloric curves for this geometry are presented altogether with the curves for the Lindemann parameter, and for the $Q_{6}$ global parameter, both as function of temperature. These curves are shown in Figure 6.

The figure shows that for icosahedra, the Lindemann parameter varies approximatelly between 0.025 and 0.16, with low values for particles with a large proportion of gold, and high values for particles with a large amount of palladium, while the order parameter $Q_{6}$ takes values close to 0.18 at low temperatures, to drop down to values very close to zero after melting, as expected. The figure also shows that the caloric curves behave in a similar manner irrespective of the size of the nanoparticles. When the melting temperatures are compared against cuboctahedral nanoparticles of the same sizes (see Figure 4), it is found that melting in icosahedra occur at higher temperatures. It is worth to remind here that icosahedra cannot be built directly by cutting a fcc geometry, and the structure is made by concentric icosahedral layers, such that the distance between first neighbors from two adjacent layers is not equal to the equilibrium distance within a same layer; this structure in concentric atomic shells arranged in an icosahedral pattern asures that the most outer shell is highly coordinated to minimize surface energy; this lower energy configuration makes icosahedral nanoparticles more resistant to thermal fluctuations, and thus the solid-to-liquid transition gets shifted to higher temperatures.

**Figure 7:**
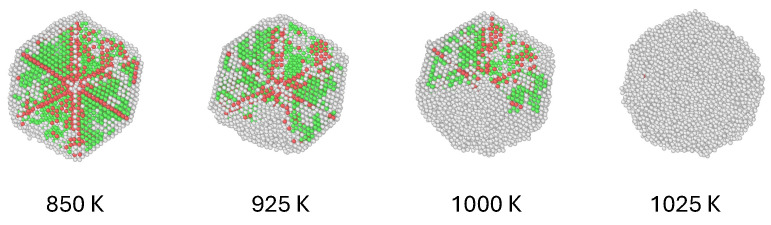
Sequence of configurations for a ${\mathrm{Au}}_{50}-{\mathrm{Pd}}_{50}$ 12,431-atom icosahedral nanoparticle as temperture is increased until reaching the melting transition (1025 K). Colors are associated with the kind of local ordering: fcc (green), hcp (red), and liquid-like (white).

It can also be noted in Figure 6 that the abrupt change in configurational energy that signals the melting transition is softer in icosahedra than in the other two geometries. This could be related at least partly to the fact that the outer layers, more uniform in their structure than those of cuboctahedra, form a more even liquid-like shell surrounding the solid core previous to melting. Nevertheless, structural analysis by DXA show that, previous to melting, when the caloric curves start deviating considerably from linearity, the common trend to all the icosahedra nanoparticles is that premelting at the surface is followed by the disordering of large portions of several of the tetrahedra separated by twins, in a way even more defined than in the case of decahedra. We found that this trend is not dependent on composition. To illustrate this, we used the case of the largest ${\mathrm{Au}}_{50}-{\mathrm{Pd}}_{50}$ icosahedral nanoparticle. Figure 7 shows a sequence of configurations close to the melting transition (1025 K for this size and composition). Just as in figure The spheres representing atoms are colored by kind of structure, just as we did in Figure 5. From the sequence, it can be noted that the first tetrahedron melts at 925 K, coinciding with the temperature at which both the caloric curve and Lindemann parameter begin to shift from their previous range. This is particularly important from experimental purposes, since, as it was discussed before, direct detection of the melting of individual tetrahedra by STEM imaging can be quite difficult.

### 3.4. Melting Lines

**Figure 8:**
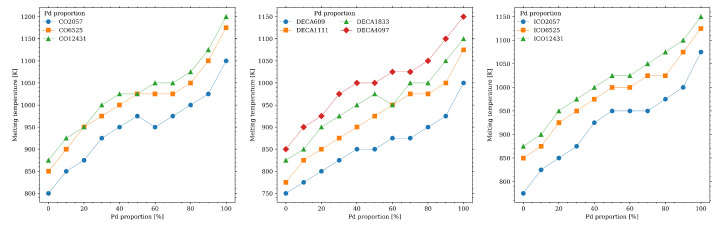
Melting temperature as function of Pd concentration for (**left**) cuboctahedral (**center**) decahedral (**right**) icosahedral Au-Pd particle.

The overall results may be summarized by building the melting curves for all the nanoparticles considered here; these results are shown in Figure 8. The curves were built using of measurements of the global order parameter $Q_{6}$, with an additional input from the caloric curves an Lindemann parameter when there was uncertainty in the value of $T_{m}$. It is worth to remind here that temperature was varied in increments of 25 K, and thus the curves have to be interpreted considering this resolution in temperature. Even with these limitations, the curves show that the melting is congruent at all of the compositions and geometries.

## 4. Discussion and Conclusions

We investigated the melting points of cuboctahedral, decahedral, and icosahedral AuPd nanoparticles using molecular dynamics simulations. We used a version of the Gupta potential, a many-body empirical potential, with a parametrization chosen to have good agreement with theoretical and experimental results. Several parameters and quantities were used to characterize the melting of AuPd nanoparticles with different geometries. To identify the melting temperature, the variation of potential energy was investigated, as is usual [43].

**Figure 9:**
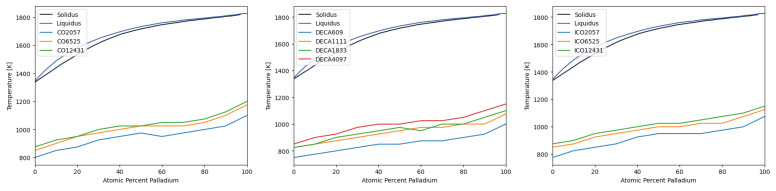
Bulk Au-Pd solidus and liquidus curves compared against the melting curves of (**left**) cuboctahedra (**center**) decahedra (**right**) icosahedra obtained from the simulations.

The melting line of the bulk AuPd alloy was compared against the obtained melting temperatures obtained from our simulations; the result is shown in Figure 9. Because of the size of the nanoparticles studied, we were unable to differentiate the solidus from the liquidus curve for our systems. The first noticeable feature is that the melting temperature is greater for the bulk alloy than what it is for the AuPd nano-alloy in the studied geometries. Dependence of size can also be noted, as expected [10,14]. We found that the kind of nanoparticle geometry—cuboctahedral, icosahedral, or decahedral—influences the melting behavior and structural stability of the AuPd nanoparticles. Cuboctahedral nanoparticles exhibit a relatively uniform melting process, driven by surface premelting, with [100] and [111] facets melting at different rates due to surface energy anisotropy. In contrast, in decahedral nanoparticles, the grain boundary junctions that separate the five tetrahedral subunits, act as regions of instability due to the strain associated with disclination defects; these boundaries serve as preferential sites for atomic diffusion, leading to an uneven melting process where one or more tetrahedral subunits melt before the entire particle transitions to the liquid phase. Icosahedral nanoparticles show the highest thermal stability, with a gradual melting initiated by surface premelting, followed by the progressive disordering of internal tetrahedral subunits.

In the three geometries, there is an atomic rearrangement, mainly at the surface of the particle of the particle, that lowers the value of the order parameter below that characteristic of a FCC structure, even before reaching the melting temperature. This is mainly due to the repositioning of low coordination atoms at the surface; since the ratio of the number of atoms at the surface against the total number of atoms is larger as the particle is smaller, the effect of this rearrangement in the values of $Q_{6}$ is more evident in the smallest particles (first row in the Figure 2, Figure 4 and Figure 6) than in the largest ones (third row). Right before melting, the particles still resemble their original geometries, but the atoms at the surface are disordered to a degree that explains the drop of the orientational order parameter. This observation is consistent with the measurements of the fall of the local order parameter q6 at the surface of the particle, around 200 K before the particle melts as a whole. The effect is more pronounced in Pd-rich nanoparticles, because the higher atomic mobility and weaker Pd-Pd surface bonding (due to the low coordination) result in earlier disordering at lower temperatures.

Regarding the use of STEM simulated micrographs in our MD studies, the contrast variations observed in the HAADF-STEM micrographs is related to differences in the ordering in atomic columns, which may correspond to the loss of crystallinity in melted regions. Even in areas where the atomic order is partially present, the intensity of the HAADF-STEM signal is influenced by partial atomic disorder, making it challenging to distinguish between well-ordered and melted regions. This highlights the value of simulated micrographs in interpreting experimental STEM images, where localized melting effects could otherwise be misidentified as differences in thickness. We believe that a study directed to investigate this particular matter may be useful in the interpretation of real HAADF-STEM micrographs of nanoalloys.

We believe that this work represents an effort that exemplifies the way that the use of atomistic simulations in conjunction with HAADF-STEM simulations may contribute to make appropriate interpretations of experimental micrographs, where the the local heating effect of the electron beam in small nanoparticles would be otherwise unnoticed. Extension of this kind of analysis to high entropy alloys at the nanoscale may also be an efficient tool for the investigation of the interplay of composition, geometry, and local structure in the thermal response of these systems.

## Data Availability

The original contributions presented in this study are included in the article/Supplementary Materials. Further inquiries can be directed to the corresponding author.

## Supplementary Materials

The following supporting information can be downloaded at: https://www.mdpi.com/article/10.3390/ma18051054/s1, File S1: LAMMPS script for the heating process.
